# Toxicity and Immunogenicity in Murine Melanoma following Exposure to Physical Plasma-Derived Oxidants

**DOI:** 10.1155/2017/4396467

**Published:** 2017-06-27

**Authors:** Sander Bekeschus, Katrin Rödder, Bob Fregin, Oliver Otto, Maxi Lippert, Klaus-Dieter Weltmann, Kristian Wende, Anke Schmidt, Rajesh Kumar Gandhirajan

**Affiliations:** ^1^ZIK plasmatis, Leibniz Institute for Plasma Science and Technology (INP Greifswald), Felix-Hausdorff-Str. 2, 17489 Greifswald, Germany; ^2^ZIK HIKE, Fleischmannstr. 42-44, 17489 Greifswald, Germany

## Abstract

Metastatic melanoma is an aggressive and deadly disease. Therapeutic advance has been achieved by antitumor chemo- and radiotherapy. These modalities involve the generation of reactive oxygen and nitrogen species, affecting cellular viability, migration, and immunogenicity. Such species are also created by cold physical plasma, an ionized gas capable of redox modulating cells and tissues without thermal damage. Cold plasma has been suggested for anticancer therapy. Here, melanoma cell toxicity, motility, and immunogenicity of murine metastatic melanoma cells were investigated following plasma exposure in vitro. Cells were oxidized by plasma, leading to decreased metabolic activity and cell death. Moreover, plasma decelerated melanoma cell growth, viability, and cell cycling. This was accompanied by increased cellular stiffness and upregulation of zonula occludens 1 protein in the cell membrane. Importantly, expression levels of immunogenic cell surface molecules such as major histocompatibility complex I, calreticulin, and melanocortin receptor 1 were significantly increased in response to plasma. Finally, plasma treatment significantly decreased the release of vascular endothelial growth factor, a molecule with importance in angiogenesis. Altogether, these results suggest beneficial toxicity of cold plasma in murine melanomas with a concomitant immunogenicity of potential interest in oncology.

## 1. Introduction

With over 70,000 new incidences and 10,000 deaths annually in the U.S. alone, melanoma is a highly prevalent type of cancer [1]. Advances have been made in melanoma therapy in the past decade but stage IV survival of nonresponder patients is still poor [2]. This owes partly to melanomas having the highest mutational burden but at the same time also having the most neoantigens among all types of cancers in humans [3]. Similar to other types of cancer, the majority of patients die due to metastasis spreading throughout the body [4]. This requires an understanding of cellular behavior and motility in response to therapy [5]. BRAF, NRAS, and MEK inhibitors improved end-stage melanoma patient survival [6]. Melanoma immunotherapy with anti-PD-(L)1 and anti-CTLA-4 antibodies further revolutionized therapy by abolishing cancer immunosuppression of tumor-specific T cells [7]. Moreover, increased immunogenicity correlates with CD163^+^ cellular infiltrate that in combination with the number of FOXP3^+^ cells is a predictor of survival [8]. Immunogenic cell death (ICD) is hallmarked by expression of calreticulin [9] which makes tumor cells visible to the immune system [10]. Of note, mitochondrial-derived reactive oxygen species (ROS) and reactive nitrogen species (RNS) and subsequent oxidative events seem to contribute to some molecular ICD events following chemo- and radiotherapy [11].

Cold physical plasma is an ionized gas and potently generates ROS and RNS of different kinds [12]. Several studies indicated the involvement of mitochondria in plasma-mediated cancer cell death, underlining the notion that exogenous as well as endogenous reactive oxygen species may be at work [13–15]. Accordingly, cold plasma has been suggested as an interesting tool in skin cancer [16] and generally in tumor therapy [17] before. The first work also pointed at the plasma's potential to involve immunogenic cell death [18]. Interestingly, antioxidants were shown to enhance metastatic spreading in a murine melanoma model [19].

Hence, the effects of cold plasma-derived oxidants on cell motility, cytotoxicity, and immunogenicity were studied in murine melanoma cell line. It was found that all of these three important hallmarks of cancer were affected by exposure to plasma. These results are promising with regard to cold plasmas potentially having a future role in combination therapy in oncology.

## 2. Materials and Methods

### 2.1. Cell Culture and Plasma Treatment

Murine, metastatic B16F10 cells (ATCC CRL-6475) were maintained in Rosswell Park Memorial 1640 (RPMI1640) medium (Pan BioTech, Germany) containing 10% fetal bovine serum, 2% penicillin/streptomycin, and 1% glutamine (all Sigma, Germany). For plasma treatment in 24-well dishes (NUNC, Denmark), 5 × 10^4^ cells were added per well. For treatment in 96-well plates (NUNC), 1 × 10^4^ cells were given to each well. Cells were allowed to adhere overnight. As plasma source, an atmospheric pressure argon plasma jet (kINPen 11) was utilized. This plasma primarily acts via ROS and RNS and is not genotoxic [20, 21]. The device is technically similar to the kINPen MED that received accreditation as medical product for skin disease. Argon gas (99.999% pure; Air Liquide, France) was used to ignite the plasma at a frequency of about 1 MHz [22]. The jet was hovered over the cells for the indicated time using a computer-programed *xyz*-table (CNC, Germany).

### 2.2. Redox-Sensitive Probe and High-Content Imaging

Cells were loaded with CM-H_2_DCF-DA (Thermo Fisher, USA) and treated with plasma or were left untreated. Fluorescent microscopy (Observer Z.1; Zeiss, Germany) was employed to image dye fluorescence facilitated by intracellular oxidases. Quantification of the cells' mean fluorescent intensities was facilitated using Fiji software. Metabolic activity was assessed by incubating the cells with 7-hydroxy-3H-phenox-azin-3-one-10-oxide (resazurin; Alfa Aesar, USA). Subsequently, fluorescent resorufin was quantified using a microplate reader measuring at *λ*_ex_ 535 nm and *λ*_em_ 590 nm (Tecan, Switzerland). To assess viability visually, propidium iodide (PI; Sigma) was added, and cells were imaged with a high-content imaging device (Operetta CLS; Perkin-Elmer, Germany) at different time points following treatment. For each time point, the total number of cells was quantified using digital phase contrast (DPC), and the number of PI positive were expressed as percent of that. In a similar manner, the total growth area was calculated for different time points following plasma treatment. DPC was used to identify cells, and only viable cells were included in the analysis before normalization to untreated control was calculated. To quantify cell motility, cells were plasma-treated and subsequently imaged every 20 min over three hours. Only PI^−^ cells (identified using DPC) were tracked. Mean displacement per cell over time was calculated. To identify mean nuclear area per cell for each treatment, B16F10 melanomas were fixed with PBS/PFA (4%, Sigma), permeabilized with 0.1% Triton X 100 (Sigma), and stained with DAPI. Nuclei area was quantified using automated image analysis. A similar protocol was applied to quantify cytosolic mean fluorescence intensity of zonula occludens 1 (ZO1 antibody; AbCam, UK) protein. The cytosolic area was determined using DPC, and the nuclear area was subtracted from that. Data analysis was performed using Harmony 4.5 software (PerkinElmer).

### 2.3. Real-Time Deformability Cytometry

Real-time deformability cytometry (Zellmechanik, Germany) allows analyzing the mechanical properties of cells with a throughput of up to 1000 cells per second [23]. The setup is built around an inverted microscope (Zeiss Observer, Germany) having a PDMS-based microfluidic chip assembled on the translation stage. One to two hours after plasma treatment, the cell suspension was driven through the central constriction of the chip by a syringe pump (Nemesys; Cetoni, Germany) at different flow rates between 0.16 *μ*l/s and 0.32 *μ*l/s. Inside the constriction, cell deformation was induced by a laminar flow profile and recorded by a high-speed camera (MC1362; Mikrotron, Germany) at 2000 frames per second. Image analysis was done on the fly enabling the quantification of size and deformation for each cell. For sample preparation, cells were centrifuged and resuspended in PBS containing 0.5% (*w*/*v*) methylcellulose to a final concentration of 10^6^ cells per ml. For each sample, at least 5000 events were acquired. An analytical model calculating the hydrodynamic flow profile around a cell inside the channel allows to link cell deformation to material properties [24] and derivation of the cells' Young's modulus [25]. Here, cell deformation is calculated from

(1) $$\begin{array}{c} d=\frac{1-\left(2\surd \pi A\right)}{1}, \end{array}$$

where *A* represents the area of the cell and *l* the perimeter. Statistical analysis was based on linear mixed models, which separates random effects, for example, biological variability, from fixed effects, for example, treatment of cells.

### 2.4. Cell Surface Marker Expression

Cells were detached using accutase (BioLegend, UK) and incubated with monoclonal antibodies directed against MHC I allophycocyanin (BioLegend), melanocortin receptor 1 (MC-1R) fluorescein isothiocyanate (Bioss, USA), and calreticulin (CRT) Alexa Fluor 647 (AbCam, UK). Cells were washed and resuspended in PBS containing 1% bovine serum albumin (Sigma) and 4′,6-diamidino-2-phenylindole (DAPI; Sigma). Cellular properties were acquired using multicolor flow cytometry (CytoFlex; Beckman-Coulter, Germany). Only viable (DAPI^−^) cells were included for the analysis of cell surface marker mean fluorescent intensities. Kaluza 1.5a software (Beckman-Coulter) facilitated data analysis.

### 2.5. Vascular Endothelial Growth Factor

Cell culture supernatants were stored at −80 °C until analysis. Concentrations of vascular endothelial growth factor (VEGF) were assessed using an enzyme-linked immunosorbent assay (ELISA) kit (BMS619-2) according to the vendor's instructions (eBioscience, Germany).

### 2.6. Statistics

Graphing and statistical analysis was performed using prism 7.02 (GraphPad Software, USA). Mean and standard errors were calculated and analyzed according to statistical methods given in the figure legends. Groups or treatments differing significantly were marked with asterisks (^∗^*p* < 0.05; ^∗∗^*p* < 0.01; and ^∗∗∗^*p* < 0.001).

## 3. Results

### 3.1. Plasma Oxidized Melanoma Cell and Decreased Metabolic Activity and Viability

**Figure 1:**
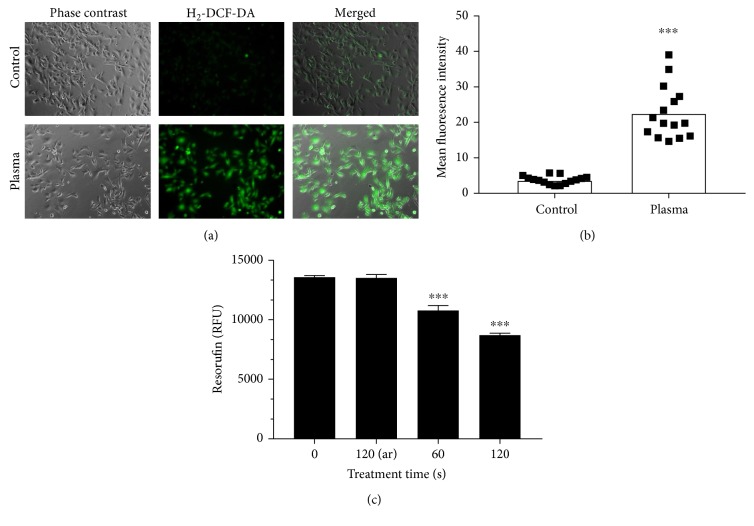
Oxidation and metabolic activity. (a) B16 melanoma cells were loaded with H_2_-DCF-DA and subjected to plasma treatment (120 s) or not. (b) Quantification of mean fluorescence intensities of the cells. (c) Mean fluorescence intensity of resorufin representative for cellular metabolic activity. Data are one representative (a, b) and mean + S.E. (c). Statistical analysis was carried out using *t*-test.

**Figure 2:**
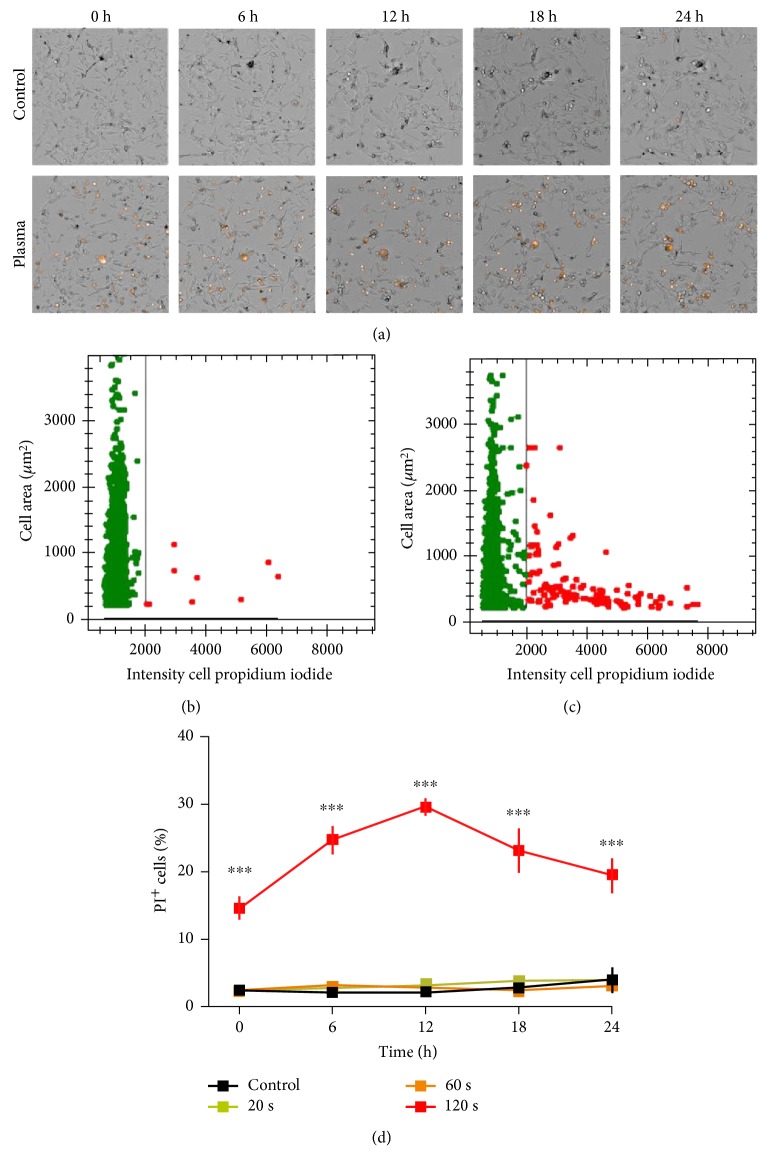
Cell death. (a) Representative bright field and PI overlay images of control (upper row) and plasma-treated (120 s, lower row) cells at different time points following exposure. (b) Representative dot plot of control cell area versus PI intensity per cell. (c) Representative dot plot of plasma-treated (120 s) cells and their area versus PI intensity per cell. Image quantification and normalization of PI^+^ cells of all cells per field of view. Data are presented as mean ± S.E. of nine replicates. One representative of three independent experiments is shown. Statistical analysis was performed using *t*-test.

Cold physical plasma generated many different kinds of oxidants. In cells loaded with H_2_-DCF-DA, plasma treatment increased total fluorescence in B16 melanoma cells compared to untreated controls (Figure 1(a)). Quantification of individual cellular fluorescence yielded a significantly enhanced mean fluorescence intensity (Figure 1(b)). To assess the cytotoxic effects, metabolic activity was assessed 3 hours after plasma treatment. Exposure to plasma for 60 s or 120 s but not 120 s of argon gas alone significantly decreased metabolic activity (Figure 1(c)). Subsequently, plasma-treated and control cells were imaged at different time points following in presence of PI indicative for cell membrane damage (Figure 2(a)). Utilization image-based quantification algorithms and the total number of cells as well as their mean fluorescence intensity of PI were determined (Figure 2(b)). Quantification and normalization to total cells revealed a significant increase in terminally dead cells in samples that had received 120 s of treatment (Figure 2(c)). Peak percent of dead cells was measured 12 h after treatment with a decrease after that. Altogether, plasma oxidized melanoma cells and decreased their metabolic activity by inducing terminal cell death.

### 3.2. Plasma Affected Cell Growth, Motility, and Biomechanical Properties

**Figure 3:**
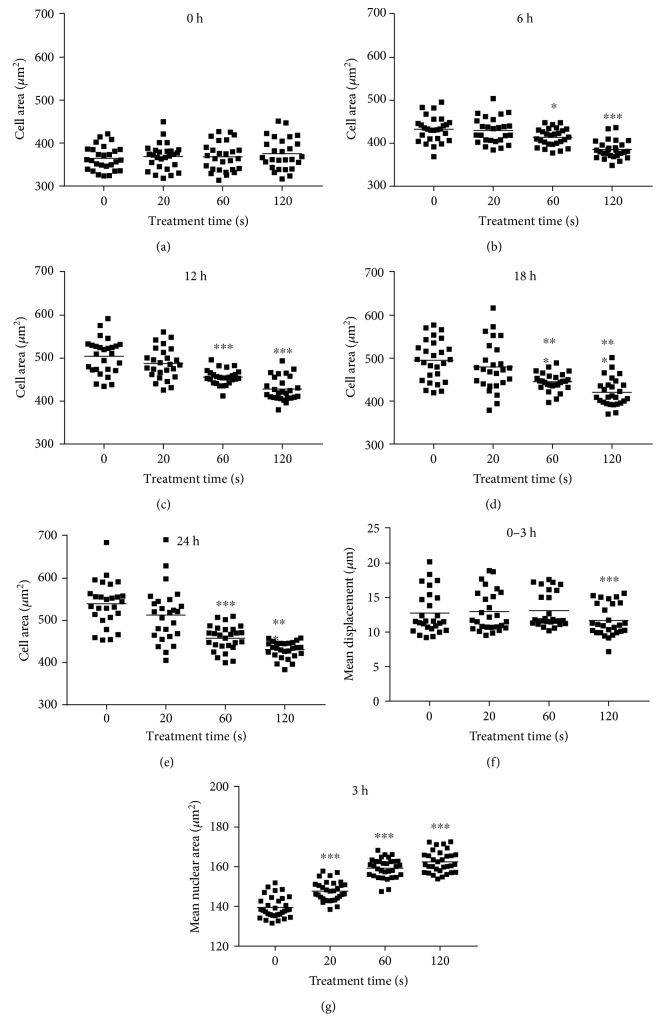
Melanoma growth kinetic and migration. (a–e) Total cell growth area per field of view was determined after several time points following plasma treatment and using automated image quantification. (f) PI^−^ melanoma motility as a function of mean cell displacement was calculated using time-lapse microscopy over 3 h and kinetic tracking algorithms. (g) Mean nuclear area of cells 3 h after plasma treatment. Data are presented as the mean of 9 replicates of each of the three independent experiments resulting in about 2000 single cells per treatment and time point. Statistical analysis was carried out using one-way ANOVA.

**Figure 4:**
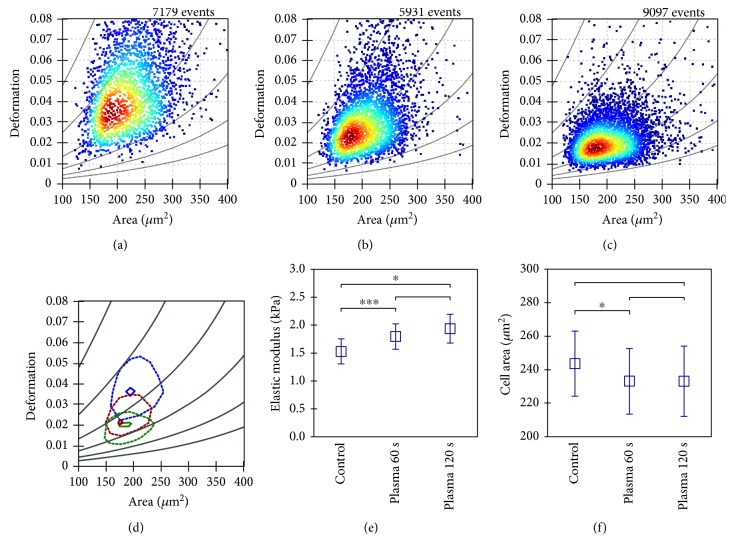
Real-time deformability cytometry. (a) Real-time deformability cytometry data of a control sample was compared to cells after 60 s (b) and 120 s (c) plasma treatment. (d) The 50% and 90% density lines of each population are given for control (blue) and plasma-treated (red 60 s, green 120 s) cells. (e) After plasma treatment, melanoma cells revealed a significant increase in Young's modulus whereas individual cell area (f) was nearly unaffected. Measurements have been carried out in a 30 *μ*m channel at a frame rate of 2000 fps. Data shown are one representative (a–c) or mean (d) ± S.E. (e, f) of three independent experiments.

**Figure 5:**
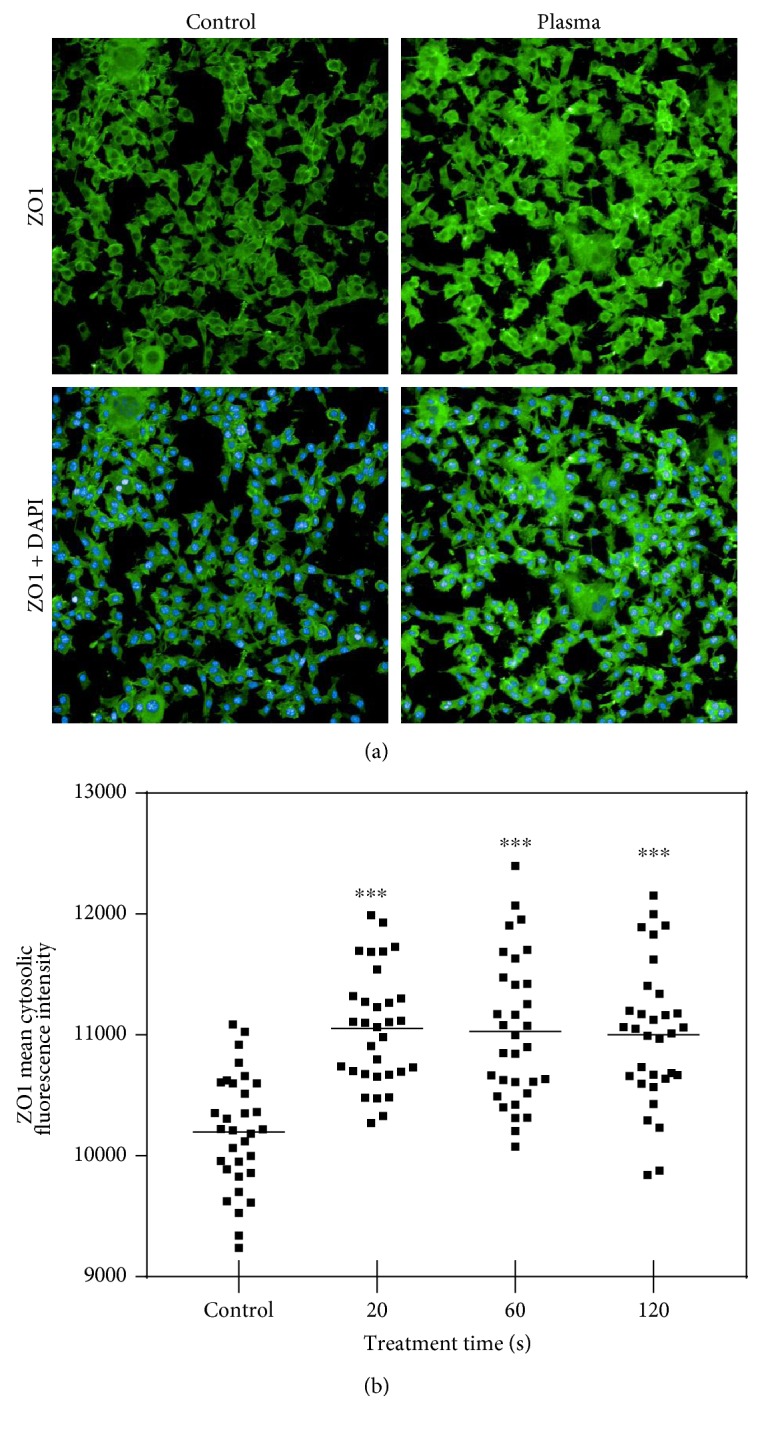
ZO1 expression. (a) Representative images of ZO1 and ZO1/DAPI immunofluorescence of control and plasma-treated (120 s) murine melanomas 3 h after exposure. (b) Quantification data are presented as mean of 8 replicates of each of the four experiments. Statistical analysis was performed using one-way ANOVA.

Next, total cell area and cell motility was assessed in PI^−^ (viable) cells. Total cell area was quantified at different time points postplasma treatment. Immediately following the treatment, the cell area was not affected (Figure 3(a)). By contrast, 60 s and 120 s of plasma treatment gave a significantly reduced cell area (Figures 3(b), 3(c), 3(d), and 3(e)). In the 120 s treated samples, the area was almost similar within the first hour (Figure 3(a)) compared to 6 h (Figure 3(b)) after treatment. This was not the case with all other samples where an increased cell area was observed. This suggested that also the viable cells were halting proliferation and possibly migration. Thus, the mean displacement of each viable cell was determined over three hours in controls and plasma-treated cells. In the 120 s plasma-treated sample, total displacement per viable cell was significantly decreased (Figure 3(f)). Concomitantly, mean nuclear area was significantly enlarged, arguing for cell cycle arrest. Both facts indicate decelerated cell motility, which is linked to biomechanical properties. Therefore, real-time deformability cytometry was performed in murine B16F10 control (Figure 4(a)) melanoma cells as well as following exposure to 60 s (Figure 4(b)) and 120 s (Figure 4(c)) of plasma treatment. After 60 s of plasma treatment, the median deformation and cell area changed from *d* = 0.041 to *d* = 0.027 and *A* = 216.6 *μ*m^2^ to *A* = 202.6 *μ*m^2^, respectively. A further reduction in median deformation to *d* = 0.02 was seen after 120 s of plasma treatment. This is summarized in Figure 4(d) by comparing the contour lines of each population. Overlay contour lines of each population clearly marked differences between all samples (Figure 4(d)). Application of an analytical model [25] allows for calculation of cellular properties. Significant differences were obtained between plasma-treated and control samples (Figure 4(e)). Sixty seconds of plasma treatment led to a significant increase in Young's modulus from 1.53 ± 0.22 kPa to 1.79 ± 0.23 kPa. Plasma exposure of 120 s resulted in an even higher elastic modulus of 1.94 ± 0.26 kPa. This alteration in mechanical properties was accompanied by a small decrease in cell area from 243.6 ± 19.4 *μ*m^2^ to 233.1 ± 19.1 *μ*m^2^ (Figure 4(f)). An integral part of tight junction formation, membrane-associated ZO1 expression is inversely linked to motility. Immunofluorescence staining gave an increase in cytosolic staining of ZO1 3 h following plasma treatment (Figure 5(a)). This increase was significant even with short, nontoxic plasma treatment times (Figure 5(b)). We also stained melanoma cells with antibodies targeted against occludin and e-cadherin but staining was weak, and changes upon plasma treatment were not observed (data not shown). Altogether, plasma decreased melanoma cell growth, motility, and deformability together with an increased ZO1 expression.

### 3.3. Plasma Increased the Immunogenicity and Decreased VEGF Release in Melanomas

**Figure 6:**
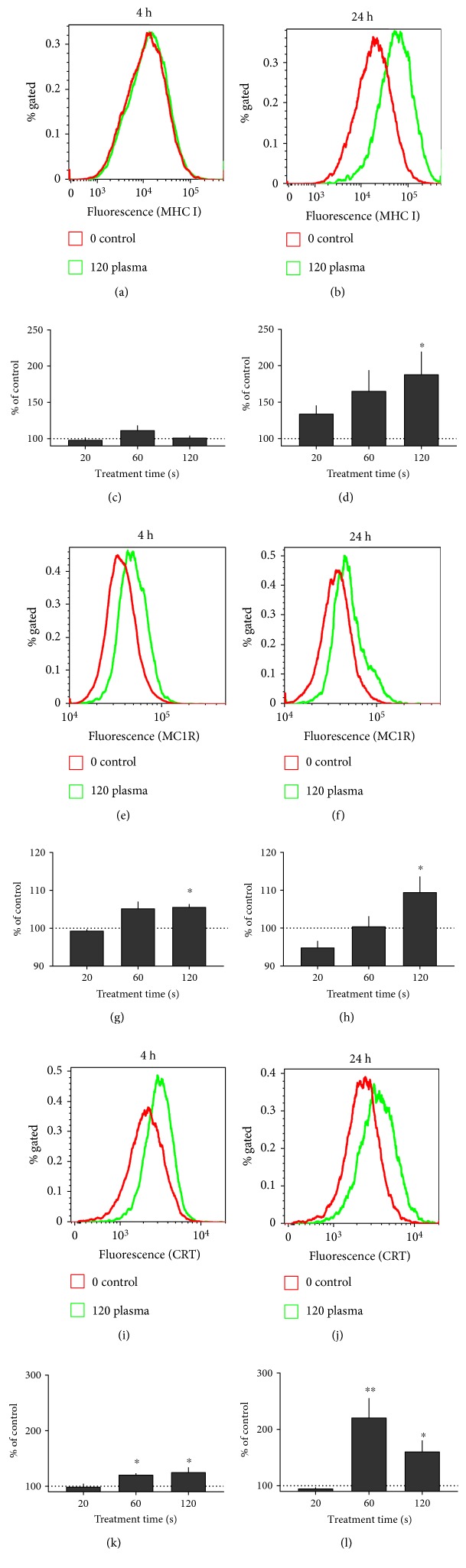
Cell surface marker expression. Cell surface marker expression of B16 melanoma cells 4 h (images on the left) or 24 h (images on the right) after plasma treatment. Representative overlay histograms of (a, b) MHC I, (e, f) MC1R, and (i, j) CRT are given. Quantification and normalization mean fluorescence intensity of each surface marker is shown for (c, d) MHC I, (g, h) MC1R, and (k, l) CRT. Data are presented as mean + S.E. of 3-4 independent experiments. Statistical analysis was performed using *t*-test.

**Figure 7:**
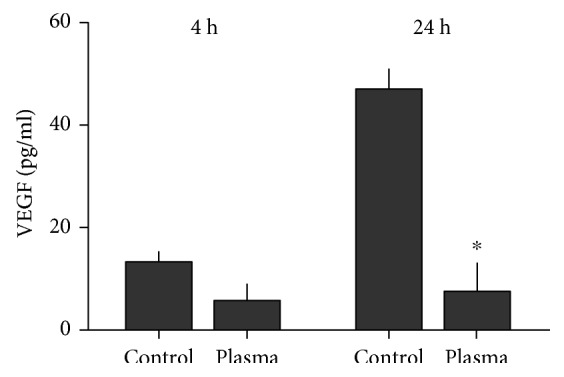
VEGF release. Cell culture supernatants were harvested 4 h and 24 h after plasma treatment, respectively. The concentration of VEGF was determined via ELISA. Data are presented as mean + S.E. of three independent experiments. Statistical analysis was performed using *t*-test.

Successful melanoma therapy is strongly linked to immunomodulation. Therefore, the expression of several cell surface molecules was investigated 4 h and 24 h following plasma treatment. Representative overlay histograms are given for each protein and time point (Figure 6). With MHC I, a significant increase was not seen after 4 h (Figure 6(c)) but was seen after 24 h (Figure 6(d)) in 120 s plasma-treated samples. This pointed to an increase in antigen presentation promoting immune recognition. For MC-1R, an important receptor in melanocyte biology, a subtle but significant increase was seen 4 h (Figure 6(e)) and 24 h (Figure 6(f)) after plasma treatment. Calreticulin (CRT) is the key molecule in immunogenic cell death (ICD). CRT was significantly increased after both 4 h (Figure 6(i)) as well as 24 h (Figure 6(j)) following exposure to plasma. Angiogenesis is important for tumor blood supply. VEGF—being a major molecule in the formation of blood vessels—was significantly decreased (Figure 7) 24 h after plasma treatment. In our hands, VEGF decrease was greater than cell viable decrease (see Figure 2(d)).

## 4. Discussion

Cold plasma treatment affected melanoma cell viability, motility, and immunogenicity. Immunogenic properties such as therapy-induced upregulation MHCI and CRT are vital for antitumor immune responses [26]. MHCI is vital for presentation of endogenous and potentially tumor-specific (neo) antigens to cytotoxic T cells [27]. Vice versa, tumor cell elimination with high MHCI expression favors the generation of MHCI^low^ cancer cells, especially in metastasis [28]. Therefore, upregulation of MHCI is viewed as a therapeutic goal in many types of tumors [29–31]. Similar to plasma, photodynamic therapy uses oxygen radicals and was shown to restore MHCI expression in human glioma [32]. Along similar lines, radiation upregulates MHCI expression in the breast [33], lung [34], and colon cancer [35]. Similar to downregulated MHCI, elevated levels of VEGF are also important for tumorigenesis [36]. We saw a drastic decrease in VEGF release likely owing to cellular toxicity. Nonetheless, an Akt-mediated increase in intracellular oxidants was previously linked to enhanced VEGF release [37]. Hence, VEGF release might be redox controlled, and its reduction would be therapeutically desired [38]. By enhancing immunogenicity, also CRT correlates with favorable prognosis for patients with, for example, lung cancer [39], gastric cancer [40], and leukemia [41]. CRT on melanoma cells was also involved in dendritic cell vaccination in melanoma patients, although cell death was found to be dispensable for that effect [42]. Exogenously added CRT also potentiates the immunogenicity of melanomas in patients [43]. A CRT fusion-protein added to B16 cells evoked an antitumor immune response in mice [44]. Intriguingly, therapeutic intervention associated with upregulation of CRT involves the generation of reactive species [45–47].

Cold physical plasma expels reactive molecules known to be important in redox biology and medicine [48]. In contrast to intracellular generation with PDT and radio- or chemotherapy, plasma-generated species are applied exogenously from ambient air to cells and tissues [49]. kINPen plasma-generated reactive molecules include for example peroxynitrite, hydrogen peroxide, and hydroxyl radical [50–52]. Today's view is that most oxidative events in cells are translated by redox enzymes and thiol switches in transcription factors which then guide the cellular response [53]. For example, we previously identified activator protein 1 (AP1) family members such as FOSB and JUND in plasma-treated blood cancer cell lines to be crucial [54]. Both factors are redox-regulated [55], and their expression was dysregulated in metastatic melanoma [56]. This makes AP1 a crucial regulator of cell regulation and death [57], as observed in our study with a decrease in metabolic activity and cell cycle arrest and increase in terminally dead cells. Interestingly, JUN proteins are involved in melanoma migration [58].

Cell mechanics is a major regulator and indicator of cell function and motility [59]. The main structural component linking function to mechanical properties is the cytoskeleton consisting of filamentous actin, microtubules, and intermediate filaments. For migration, cells require to alter their morphology, which is controlled by the cytoskeleton on a molecular and the emerging mechanical properties on a cellular scale [60]. In real-time deformability cytometry, an increase in elastic modulus after plasma treatment was observed. This effect could be originated from an alteration in actin polymerization subject to redox control [61], which is also supported by the retarded migration of the cells. This is in agreement with an earlier study on fibroblasts where a direct correlation between cell elasticity and migration was shown [62]. Enhanced cell motility and therefore invasiveness correlates with increased cytosolic ZO1 protein whereas noninvasive breast cancer cells showed elevated ZO1 in the cell membrane [63]. In pancreatic cancer cells, however, membrane-associated ZO1 was supporting invasiveness [64]. In our work, we saw an increase of ZO1 not only in the cytosolic fraction but also visually in the cell membrane. This implicates a de novo translation of ZO1 proteins in melanoma cells and not necessarily its specific translocation from the membrane to the cytosol. Underlining this idea, de novo generated ZO1 in breast cancer cells was previously shown to be present in the cytosol as well as to translocate to the cell membrane [65]. Another report describes the association of melanoma ZO1 with adherence junctions of nonepithelial cells such as fibroblasts instead of tight junctions [66]. The authors concluded that knockdown of ZO1 suppresses melanoma invasiveness. Similarly, an upregulation of MC1R increases B16F10 melanoma motility [67]. Yet, the authors transfected MC1R and induced an about twentyfold increase. By contrast, MC1R upregulation after plasma was only 1.1-fold. The main function of MC1R is to control skin and hair pigmentation via eumelanin production [68]. MC1R is generally upregulated in melanoma cells [69]. This is used for therapeutic purposes to deliver target drugs into the cells, and pro-oxidant therapies such as PDT have been successfully employed in this strategy to increase survival in experimental animal models [70].

The utilization of only one cell line limits the specificity and/or generalization of our results that should be compared to nonmalignant melanocytes and confirmed in other cancer cell lines. Specifically, the relevance of our findings may increase if human cancer cells would be similarly affected. In addition, it would be valuable to identify the effects of other types of plasma sources in this model.

In summary, it was demonstrated that treatment of murine metastatic melanoma cells with cold physical plasma-derived oxidants exerted cytotoxic effects, decreased cell motility, and increased their immunogenicity. Animal models need to provide evidence whether plasma-inactivated melanoma experiences a vaccine-like immunogenic cell death (ICD) which would make plasma therapy an interesting new tool in oncology.
